# Platelet Extracellular Vesicles Loaded Gelatine Hydrogels for Wound Care

**DOI:** 10.1002/adhm.202401914

**Published:** 2024-10-25

**Authors:** Florence Back, Alexandre Barras, Ariunjargal Nyam‐Erdene, Jen‐Chang Yang, Sorin Melinte, José Rumipamba, Thierry Burnouf, Rabah Boukherroub, Sabine Szunerits, Er‐Yuan Chuang

**Affiliations:** ^1^ Université de Lille CNRS Université Polytechnique Hauts‐de‐France UMR 8520 – IEMN Lille F‐59000 France; ^2^ International Ph.D. Program in Biomedical Engineering College of Biomedical Engineering Taipei Medical University Shuang‐Ho Campus New Taipei City 23561 Taiwan; ^3^ Graduate Institute of Nanomedicine and Medical Engineering College of Biomedical Engineering Taipei Medical University, Shuang‐Ho Campus New Taipei City 23561 Taiwan; ^4^ Université catholique de Louvain, ICTEAM Louvain‐la‐Neuve 1348 Belgium; ^5^ Graduate Institute of Biomedical Materials and Tissue Engineering College of Biomedical Engineering Taipei Medical University, Shuang‐Ho Campus New Taipei City 23561 Taiwan; ^6^ Laboratory for Life Sciences and Technology (LiST) Faculty of Medicine and Dentistry Danube Private University Krems 3500 Austria

**Keywords:** chronic wounds, hydrogel foam, platelet extracellular vesicles, skin regeneration, wound model

## Abstract

Platelet extracellular vesicles (pEVs) isolated from clinical‐grade human platelet concentrates are attracting attention as a promising agent for wound healing therapies. Although pEVs have shown potential for skin regeneration, their incorporation into wound bandages has remained limitedly explored. Herein, gelatine‐based hydrogel (PAH‐G) foams for pEVs loading and release are formulated by crosslinking gelatine with poly(allylamine) hydrochloride (PAH) in the presence of glutaraldehyde and sodium bicarbonate. The optimized PAH‐G hydrogel foam, PAH_0.24_G_37_, displayed an elastic modulus G’ = 8.5 kPa at 37 °C and retained a rubbery state at elevated temperatures. The excellent swelling properties of PAH_0.24_G_37_ allowed to easily absorb pEVs at high concentration (1 × 10^11^ particles mL^−1^). The therapeutic effect of pEVs was evaluated in vivo on a chronic wound rat model. These studies demonstrated full wound closure after 14 days upon treatment with PAH_0.24_G_37_@pEVs. The maintenance of a reduced‐inflammatory environment from the onset of treatment promoted a quicker transition to skin remodeling. Promotion of follicle activation and angiogenesis as well as M1–M2 macrophage modulation are evidenced. Altogether, the multifunctional properties of PAH_0.24_G_37_@pEVs addressed the complex challenges associated with chronic diabetic wounds, representing a significant advance toward personalized treatment regimens for these conditions.

## Graphical abstract

Gelatine‐based hydrogel foams loaded with platelet‐derived extracellular vesicles from clinical‐grade human platelet concentrates have demonstrated their effectiveness in a wound‐healing rat model, opening new perspectives for personalized care management in patients with recalcitrant wounds.

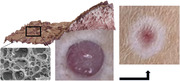

## 1 Introduction

Chronic wounds are a worldwide global healthcare burden, due to their high prevalence and recurrence frequency particularly associated with diabetes and aging.^[^ ^1^ ^]^ The complex molecular and cellular deficiencies, such as disproportionate levels of proinflammatory cytokines, proteases, and reactive oxygen species in non‐healing wounds, lead to wound closer times exceeding the normal time frame. Furthermore, most chronic wounds are accompanied by bacterial contamination involving a mixture of bacterial species notably *Staphylococcus*, *Pseudomonas*, and *Enterobacter*.^[^ ^2^ ^]^ Colonization of these bacteria, favored by the basic pH of the wound bed, together with self‐secreted extracellular polymeric substances (e.g. polysaccharides, proteins, etc.) into their environment, results in the rapid formation of biofilms.^[^ ^3^ ^]^ Such mixed communities affect wound healing due to inflammation, and the release of reactive oxygen species (ROS) as well as lytic enzymes.^[^ ^4^ ^]^ The accompanied wound exudate formation results in the degradation of growth factors and the release of protein‐digesting enzymes such as matrix metalloproteinases (MMPs).^[^ ^5^ ^]^ Furthermore, in chronic wounds, these fluids interfere with cell proliferation and are one of the reasons for delayed healing.^[^ ^6^ ^]^ While proteomics analysis of wound exudates offers a non‐invasive way of investigating wound healing, bandages capable of fluid uptake are an efficient and pain‐free manner to treat fragile wounds for therapeutic purposes. Classical wound dressings, such as bandages and gauze, absorb largely wound exudate, but at the same time result in desiccating the wound surface.^[^ ^7^ ^]^ Hydrogels with high water content are increasingly recognized as gentle tissue adhesive alternatives, even though their strong adhesion to the skin can cause pain during dressing removal.^[^ ^8^ ^]^ Hence, there is a justification to develop “intelligent” hydrogels that offer non‐irritating adhesion and enable non‐traumatic on‐demand detachment, particularly needed for treating fragile or sensitive chronic wounds.

Gelatine is well‐known for fostering skin regeneration. Gelatine is obtained by partial hydrolysis of collagen, resulting in a heterogeneous mixture ofsingle and multi‐stranded polypeptides comprising between 300 and 4000 amino acids. The structural transformation of gelatine between the single strands and triple helixes with a sol‐to‐gel transition at ≈35–40 °C makes mechanical resistance often limited over time. In addition, the amino residues in gelatine are insufficient for the fabrication of bandages with acceptable mechanical strength.^[^ ^9^ ^]^ In this work, we provide an experimental design for the preparation of mechanically robust hydrogel foams that leverage the excellent biocompatible properties of gelatine.

**Figure 1:**
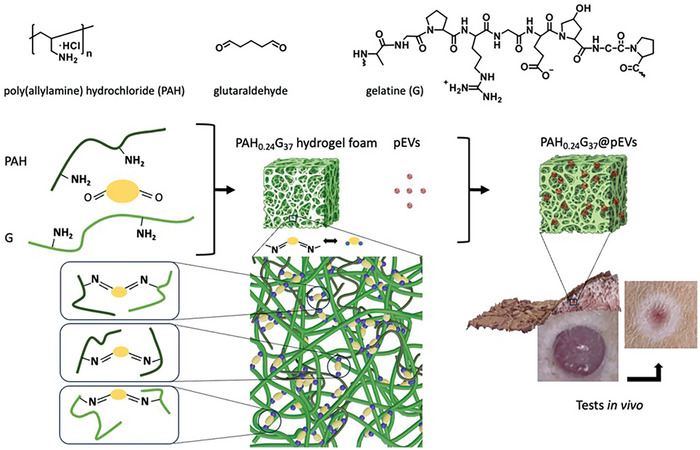
Human platelet‐derived extracellular vesicles (pEVs) loaded into gelatine‐based hydrogel foams for improved wound healing. Gelatine‐based hydrogel foams are formed by crosslinking gelatine (G) with poly(allylamine hydrochloride) (PAH) using glutaraldehyde in the presence of sodium bicarbonate(NaHCO_3_). The PAH_0.24_G_37_ hydrogel foams – with a composition of 37% w/v gelatine, 0.24% w/v PAH, 26.47 mm glutaraldehyde, 89 mm NaHCO_3_ – are loaded with pEVs (1 × 10^11^ particle mL^−1^) and applied to wounds in an in vivo diabetic rat model.

Hydrogel foams are microporous materials where the dispersed phase is a gas and the continuous one is the hydrogel.^[^ ^10^ ^]^ Microporous hydrogels typically exhibit pores between 5 and 20 µm, while super‐porous hydrogels are a subset characterized by interconnected pores.^[^ ^11^ ^]^ Furthermore, hydrogel foams can reach a porosity larger than 64% and usually display pore sizes exceeding 10 µm.^[^ ^10^ ^]^ Due to such characteristics, hydrogel foams are gaining popularity for biomedical applications where rapid and high water/liquid adsorption is essential. Sodium bicarbonate has been the basic component for the formation of hydrogel foams as CO_2_ gas is produced under acidic conditions. Porous gelatine scaffolds with an interconnected porous system could be prepared via gas foaming with the pore size depending on the amount of glutaraldehyde used for crosslinking but are in the range of the recommended pore size for skin tissue scaffolds being 20–125 µm.^[^ ^12^ ^]^ Here, we demonstrate that gelatine crosslinking in the presence of sodium bicarbonate (**Figure** **1**) results in mechanically and thermally stable PAH‐G hydrogel foams. The optimized PAH_0.24_G_37_ hydrogel foam showed excellent swelling properties and was used as scaffold to load human platelet‐derived extracellular vesicles (pEVs).

Extracellular vesicles (EVs) are spherical, subcellular structures of nanometre size enclosed by lipid bilayers, and released by multiple cell types.^[^ ^13^ ^]^ They contain a multifaceted molecular cargo of proteins, non‐coding nucleic acids, and lipids that participate in cell signaling and act as positive mediators in wound healing processes.^[^ ^14^ ^]^ Among all types of EVs, pEVs, isolated from clinically licensed allogeneic human platelet concentrates, hold the greatest promise for pragmatic translational therapeutic applications.^[^ ^13^, ^15^ ^]^ This has been lately underlined by a study of some of us on impaired wounds using gelatine‐alginate hydrogels loaded with reduced graphene oxide (rGO) and pEVs.^[^ ^14f^ ^]^ In this work, the photothermal properties of rGO were exploited to complement the therapeutic action of pEVs to reduce the expression of inflammatory biomarkers, promote angiogenesis, and enhance diabetic wound healing. In the current work, we focus entirely on the wound‐healing ability of pEVs‐loaded PAH_0.24_G_37_ hydrogel foams without any further external stimulus.

## 2 Results and Discussion

### 2.1 Formulation and Characterization of Hydrogel Foams

Gelatine, biocompatible to human tissues and mimicking the extracellular matrix, is used as scaffold base in this work.^[^ ^16^ ^]^ The mechanical behavior of gelatine‐based hydrogels depends strongly on the synthesis process as the sol‐gel transition temperature is < 35 °C. At room temperature, the polypeptide chains of gelatine assemble together in alpha helix structures and form a gel with a 3D network, while, at temperatures above its equilibrium melting temperature, these chains are broken and the gel melts. This limits consequently the utilisation of gelatine as a wound bandage material over a prolonged time. Stabilization of the gelatine network is achieved through crosslinking with PAH, a water‐soluble cationic polyelectrolyte featuring pendant primary amino groups (NH_2_) in the presence of glutaraldehyde. Furthermore, these hydrogels are fabricated in the presence of sodium bicarbonate, to give the gel a foam‐like aspect with increased porosity due to the production of CO_2_ gas and the “imprinting” of the gas bubbles in the hydrogel.

The rheological behavior of different hydrogel foams was assessed by determining the energy storage modulus (G′) and elastic modulus (G″) at different temperatures. To evaluate the optimal amount of gelatine to be used, the stiffness and the intrinsic viscosity of gelatine in absence of PAH, but constant quantity of glutaraldehyde (26.47 mm) and NaHCO_3_ (89 mmM) were determined first (see Figure S1, Supporting Information). In the absence of PAH and in the dry state, the storage modulus decreased from 25.11 kPa (37% w/v gelatine, G_37_) to 16.21 kPa (27% w/v gelatine, G_27_), 8.41 kPa (17% w/v gelatine, G_17_) with G″ of 0.94 kPa (G_37_), 0.65 kPa (G_27_) and 0.23 kPa (G_17_) at 25 °C. The hydrogel foam with 37% w/v gelatine resulted in the greatest stiffness and was used in the following.^[^ ^17^ ^]^

**Figure 2:**
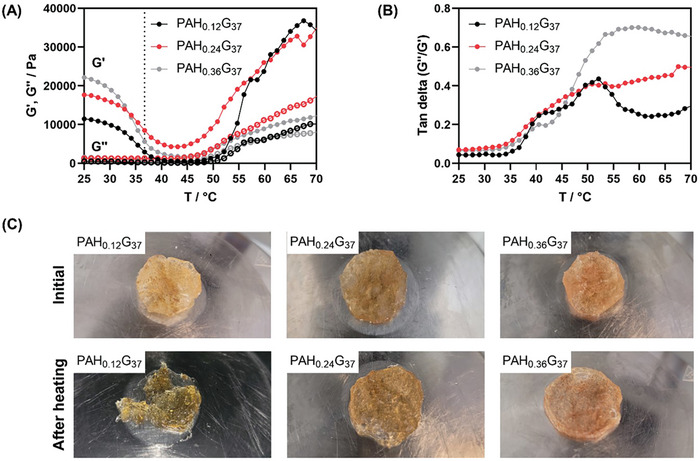
Physico‐chemical characterization of different PAH‐G hydrogel foams. (A) Storage (G’, circles) and loss moduli (G”, open circles) of PAH_x_G_37_ (x = 0.12, 0.24, 0.36) hydrogel foams composed of 37% w/v gelatine and different PAH contents: 0.12% w/v (black), 0.24% w/v (red) and 0.36% w/v (grey) in the presence of glutaraldehyde (26.47 mm) and NaHCO_3_ (89 mMM). Experimental details: 1 Hz, 1% strain, 1.5 mm gap, 1 °C min^−1^ after 3.5 h immersion in water. The dotted line at 37 °C. (B) Tan delta (G”/G’) plot as a function of temperature extracted from Figure 2A. (C) Images of PAH_x_G_37_ (x = 0.12, 0.24, 0.36) hydrogel foams before and after temperature sweep to 70 °C, (2.9 cm in diameter, 2–3 mm thick).

The storage modulus G′ values of PAH_x_G_37_, formed using 37% w/v gelatine (G_37_) upon the addition of different percentages of PAH are depicted in **Figure** **2**A. The storage moduli and thus the elastic part increased from 11.48 kPa (PAH_0.12_G_37_) to 17.69 kPa (PAH_0.24_‐G_37_) and 22.17 kPa (PAH_0.36_G_37_) at 25 °C. The loss moduli increased from 0.51 kPa (PAH_0.12_G_37_) to 1.24 kPa (PAH_0.24_G_37_) and 1.44 kPa (PAH_0.36_G_37_) (Figure 2A). At 37 °C, the hydrogel foams exhibited decreased storage moduli, suggesting limited mechanical strength on the skin; PAH_0.24_G_37_ displayed instead a significantly larger G’ at 37 °C.^[^ ^18^ ^]^ Increasing the PAH content further to 0.36% w/v (PAH_0.36_G_37_) achieved a G’ comparable with PAH_0.24_G_37_, declining strongly at higher temperature. These findings demonstrated that the presence of 0.24% w/v PAH in the final gel was optimal for skin‐based bandage. The G' value of the PAH_0.24_G_37_ hydrogel foam was comparable to the 10 kPa reported for a hyaluronic acid‐ Ɛ‐poly‐L‐lysine‐pluronic F127 hydrogel,^[^ ^19^ ^]^ or polysaccharide‐based ones (G' = 10 kPa).^[^ ^20^ ^]^ The obtained amortization factor (G”/G“) (Figure 2B) revealed that increasing the PAH content from 0.12 to 0.24% w/v increased the glass transition (T_g_) from 52 to 67 °C, while PAH_0.36_G_37_ recorded a T_g_ = ≈60 °C. Over the temperature range studied, no crossover between the storage and the loss modulus was observed, inferring that the crosslinking rate was sufficiently high for the hydrogels to remain in a rubbery state even at higher temperature. The morphology of PAH_0.24_G_37_ and PAH_0.36_G_37_ hydrogels did not change significantly after temperature sweep experiments, as seen in the photos of the gels in Figure 2C. PAH_0.12_G_37_ experienced on the other hand considerable material loss once heated up to 70 °C. To minimize eventual PAH release, the PAH_0.24_G_37_ was used in the following.

To quantify the degree of disruption of alpha chains of gelatine, the ratio of peak intensities at 1235 cm^−1^ (amide III) and 1450 cm^−1^ (proline, hydroxyproline) in the FT‐IR spectra of the hydrogel foams can be used.^[^ ^17^ ^]^ In our case, using NaHCO_3_ (89 mm), the gelatine‐only gel foams show amide III/proline ratios of ≈0.72–0.75 (data not shown), in line with the literature reporting that the presence of ionic species such as NaHCO_3_ can disrupt the alpha chains of gelatine. In the case of the hydrogel foams, ratios of ≈0.68–0.72 were determined. Indeed, PAH is itself an ionic species and the alpha chains disruption could be compensated by the presence of crosslinking and NaHCO_3_, a necessity to obtain a foam with plenty of pores.

**Figure 3:**
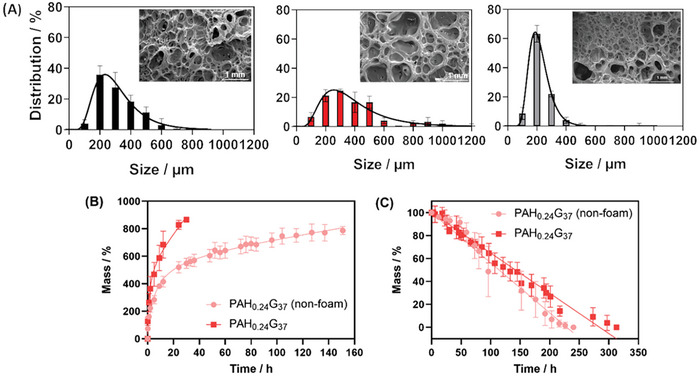
Gelatine‐based hydrogel foams. (A) Pore size distribution (gaussian fit) of PAH_0.12_G_37_ (black), PAH_0.24_G_37_ (red), and PAH_0.36_G_37_ (grey); insets: corresponding morphologies; The minimum number of pores considered is 50 with *n* = 3 replicates. (B) Swelling behavior of PAH_0.24_G_37_ in foam and non‐foam forms. Data are represented as mean ± SD, with *n* = 3 replicates. (C) Stability of PAH_0.24_G_37_ in foam (red square) and non‐foam (circles) states upon water immersion. Data are represented as mean ± SD, with *n* = 3 replicates.

Scanning electron microscopy (SEM) analysis revealed the fractal structure of the gelatine‐based hydrogel foams (**Figure** **3**A). The pore size distribution was not much affected by changes in the PAH concentration, with a maximal percentage of pores recorded at 200 nm for PAH_0.12_G_37_ (36 ± 6%) and PAH_0.36_G_37_ (63 ± 6%), and 300 nm for PAH_0.24_G_37_ (25 ± 1%). The pores inside the hydrogel fibers reinforce fibers cohesion, making the hydrogel foams superior to conventional hydrogels. The swelling properties in the water of PAH_0.24_G_37_ were investigated (Figure 3B). Dry PAH_0.24_G_37_ gel foams recorded a swelling/uptake capacity of 866 ± 17% after 30 h. This contrasts with PAH_0.24_G_37_ in non‐foam form with 549 ± 37% of water uptake capacity after 30 h. Immersion of water‐loaded PAH_0.24_G_37_ for prolonged time into water at room temperature revealed that the gel was stable over longer immersion time (Figure 3C), while PAH_0.24_G_37_ in the non‐foam state showed limited stability.

**Figure 4:**
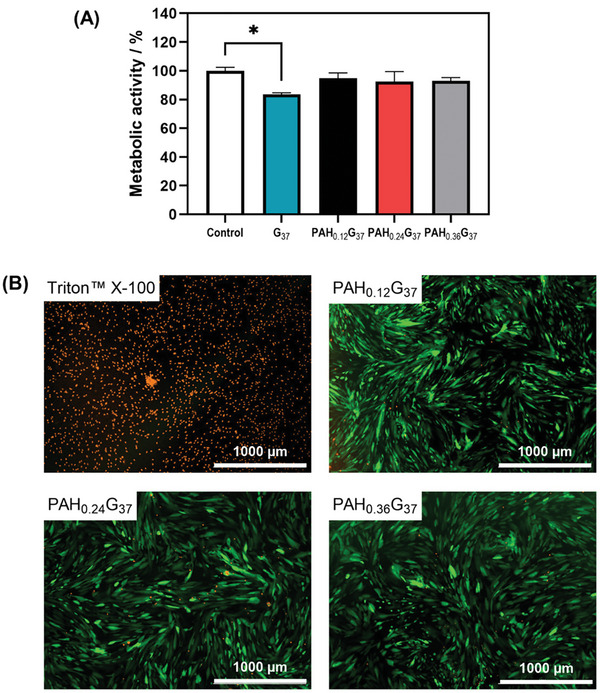
In vitro biocompatibility of gelatine and PAH_x_G_37_ hydrogel foams. (A) Metabolic activity after 24 h incubation of the pure extracts from hydrogel foams (163 ± 17 mg) with HDFCs‐adGFP (1 × 10^4^ cells/well) using the resazurin fluorescence‐based assay. Data are represented as mean ± SD, with *n* = 3 replicates, *p ≤ 0.05. (B) Dead staining images of fibroblasts after incubation for 24 h with PAH_x_G_37_ extracts and positive control using Triton^TM^ X‐100 at 0.25% v/v. The scale bar is 1000 µm. Dead or damaged cells stained using propidium iodide (orange) and GFP‐expressing HDFCs (green).

The metabolic activity of the different hydrogel foams was determined by using green fluorescent protein (GFP) expressing human adult dermal fibroblast cells (HDFCs‐adGFP). The metabolic activity was >90% for all the different hydrogel foam extracts after incubation for 24 h in complete fibroblast medium (**Figure** **4**A). The metabolic activity decreased slightly to 83% for gelatine hydrogel foams (G_37_). PAH has been shown to have a half maximal inhibitory concentration IC_50_ of ≈20–25 µg /10^5^ for human lung carcinoma cells (A549),^[^ ^21^ ^]^ with PAH concentrations ≥100 µg mL^−1^ being sublethal to Chinese hamster fibroblasts. The viability tests indicated that increasing the molecule weight of PAH via glutaraldehyde crosslinking decreased the cytotoxic nature of PAH, as witnessed by the cell viability >90% acquired for PAH‐G hydrogel foams. Indeed, PAH_0.24_G_37_ displayed favorable cell viability, in particular given the fact that PAH_0.24_G_37_ was not intended to degrade in the patient's body, but was applied as a pEVs loading matrix. Dead assays (Figure 4B) were performed in addition to visualize the ratio of live and dead fibroblasts after 24 h incubation with the PAH_x_G_37_ hydrogel foam extracts and Triton^TM^ X‐100 as a negative control. The viability of the PAH_x_G_37_ hydrogel foams correlated well with the resazurin fluorescence‐based cell viability assay (Figure 4A).

### 2.2 pEVs Loading and Release Properties of PAH_0.24_G_37_

**Figure 5:**
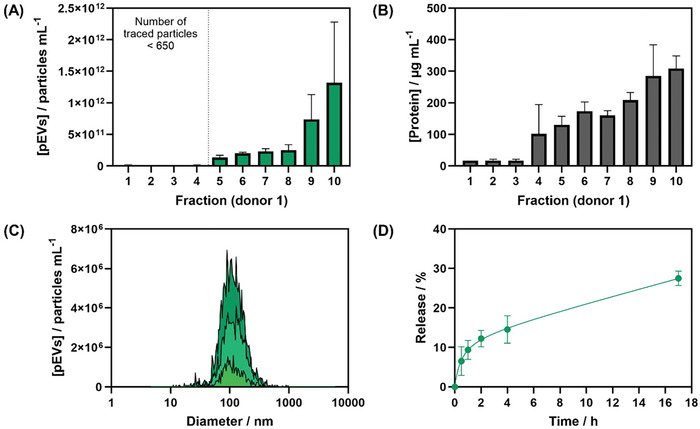
Loading/release of pEVs (donor 1) into/from PAH_0.24_G_37_ hydrogel foams. (A) concentration of pEVs as determined by NTA in the first ten fractions collected after column purification. Data are represented as mean ± SD, with *n* = 3 replicates. (B) Total protein concentration in the different fractions using bicinchoninic acid protein assay. Data are represented as mean ± SD, with *n* = 3 replicates. (C) pEVs size distribution of fraction 5 (donor 1, 3 different purifications). (D) pEVs release from PAH_0.24_G_37_ hydrogel foams loaded with (6.4 ± 0.27) × 10^10^ pEVs mL^−1^ at 37 °C. Data are represented as mean ± SD, with *n* = 2 replicates.

pEVs were isolated and purified from platelet lysates from pooled platelet concentrates using a Sepharose CL‐2B column.^[^ ^22^ ^]^ Nanoparticle tracking analysis (NTA) of the first ten eluate fractions revealed that up to fraction 4, the concentration of pEVs was >1 × 10^10^ particles mL^−1^, while fractions 5 to 10 showed a concentration of ≈1.33 × 10^11^ to ≈1.31 × 10^12^ particles mL^−1^ (**Figure** **5**A). The total protein content increased from 130 µg mL^−1^ (fraction 5) to 309 ng mL^−1^ (fraction 10) (Figure 5B). Starting from fraction 9, there was an increase in protein concentration, indicating the presence of additional free proteins besides pEVs. PAH_0.24_G_37_ hydrogel was loaded with pEVs from fractions 4 to 9 with a size distribution showing a maximum at ≈110 ± 4 nm (median of fraction 5, *n* = 3) (Figure 5C). When repeating the size‐exclusion purification step using platelet concentrates from other donors, similar results were obtained for both protein concentration and particle size distribution of the selected fractions 5, confirming the method's robustness and reliability (see Figure S2, Supporting Information).

Cryo‐EM images (see Figure S3, Supporting Information) revealed multiple vesicles in the 50–150 nm range, consistent with our previously published data using dynamic light scattering (DLS) and nanoparticle analysis with a bilayer membrane typical of EVs. The brighter background of the SCPL‐EVs images compared to the SCPL‐raw, as observed before,^[^ ^23^ ^]^ likely reflects the removal of free proteins and aggregates by SEC, as expected.

**Figure 6:**
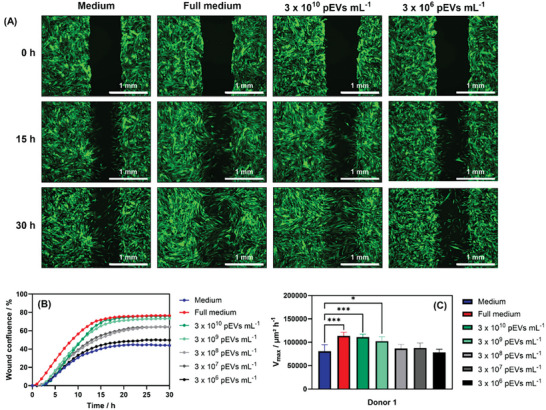
Influence of pEVs concentration on wound confluence during 30 h treatment of HDFCs‐adGFP. (A) Images of HDCFs‐adGFP cells at 3‐time intervals of treatment (0, 15, and 30 h), treated with medium, full medium as well as two different pEVs concentrations; images were taken with 2.5× (2.75× eff.) objective and GFP filter cube. (B) Wound confluence during 30 h of treatment of HDFCs‐adGFP with medium (blue), full medium (red), and five different pEV concentrations. Data are represented as mean, with *n* = 4 replicates. (C) Influence of pEV concentration on maximum wound healing rate. Values represent the mean ± SD (*n* = 4). Ordinary one‐way ANOVA with Dunnett's multiple comparisons test was used to analyze the results. Statistically significant differences were considered for *p ≤ 0.05, **p ≤ 0.01, ***p ≤ 0.001, and ****p ≤ 0.0001.

To assess the release capacity of PAH_0.24_G_37_ hydrogel foams, pEVs were fluorescently labeled with lipophilic DiO, and (6.4 ± 0.27) × 10^10^ particles mL^−1^ of these fluorescent pEVs were adsorbed totally into PAH_0.24_G_37_ at 4 °C during 3.5 h. After washing, the PAH_0.24_G_37_@pEVs hydrogel foams were immersed into PBS (1 mL) at 37 °C and the release of the DiO‐labelled pEVs was followed by NTA over the time course of 17 h (Figure 5D). The release of pEVs from PAH_0.24_G_37_ hydrogel foams exhibited a burst release during the 2 first hours and then increased over time. ≈0.6–1.1 × 10^10^ particles mL^−1^ (27.5 ± 1.8%) were released over 17 h. To validate if this concentration was sufficient for improved wound healing, the effect of the concentration of pEVs on the wound healing closure kinetics in an in vitro wound healing assay, based on fluorescence kinetic imaging, was performed using GFP expressing human adult dermal fibroblast cells. The fluorescence images of HDFCs‐adGFP cells (**Figure** **6**A) related to 30 h cell migration displayed clearly a pEV concentration‐dependent behavior. The concentration of pEVs ≥ 3 × 10^9^ particles mL^−1^ showed wound healing rates approaching that of full medium (Figure 4B). Furthermore, comparable results were obtained from pEVs collected from a different donor (see Figure S4, Supporting Information), with a clear threshold at ≈1.5 × 10^9^ particles mL^−1^.

### 2.3 In Vivo Diabetic Wound Healing Assay

**Figure 7:**
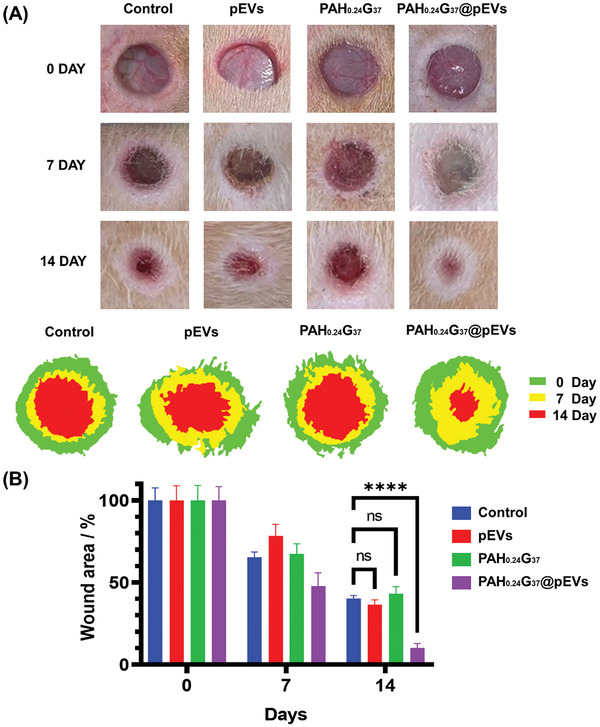
Comparative analysis of wound healing efficiency in diabetic rats. (A) Dynamic wound healing areas as calculated using ImageJ software from wounds (8 mm, sterile biopsy punch, on the back) treated with PBS (negative control), pEVs (1 × 10^11^ particles mL^−1^), PAH_0.24_G_37_ and PAH_0.24_G_37_@pEVs (loaded with 1 × 10^11^ particles mL^−1^). (B) Effectiveness of different treatments on wound healing in diabetic rats over a 14‐day period validated by the wound area (%) over time. For PBS (negative control, blue), pEVs (1 × 10^11^ particles mL^−1^, red), PAH_0.24_G_37_ (green) and PAH_0.24_G_37_@pEVs (loaded with 1 × 10^11^ particles mL^−1^, violet). For the treatment groups, the samples were topically applied to the diabetic wounds on days 0 and 7. Photographic data were collected after cleaning the wounds and before administering the formulation. All in vivo experimental procedures were independently replicated six times (*n* = 6), and the results are presented as the mean ± standard deviation (SD). Statistical analyses were conducted using GraphPad Prism software. Two‐way ANOVA (Tukey's multiple comparison test) was employed to assess statistical significance, with significance levels denoted as follows: *p ≤ 0.05, **p ≤ 0.01, ***p ≤ 0.001, and ****p ≤ 0.0001.

The efficacy of accelerated wound healing was assessed in diabetic rats, with a focus on comparing various treatments (**Figure** **7**). Figure 7A depicts a series of images documenting the progression of wound healing in diabetic rats over 14 days. The wounds, created using an 8 mm sterile biopsy punch, were treated with various formulations: PBS (as a negative control), pEVs (1 × 10^11^ particles mL^−1^), PAH_0.24_G_37_ (50 mg), and PAH_0.24_G_37_@pEVs (50 mg and 1 × 10^11^ pEVs particles mL^−1^). The wound healing performance of PAH_0.24_G_37_@pEVs (loaded with 1 × 10^11^ pEVs particles mL^−1^) was significantly more effective than treatments using pEVs of the same concentration and PAH_0.24_G_37_ alone. After 14 days of treatment, ≈40% of the wound area remained open in the PBS negative control group, evidencing inadequate closure. In contrast, the group treated with PAH_0.24_G_37_@pEVs achieved complete closure of the wound area within the same timeframe. Figure 7B quantitatively analyses the effectiveness of different wound healing treatments by plotting the wound area percentage over time. Wounds treated with PAH_0.24_G_37_@pEVs (violet bars) exhibited the most rapid and complete wound closure compared to all other treatments. By day 14, wounds treated with PAH_0.24_G_37_@pEVs were completely closed, in stark contrast to the PBS‐treated wounds (blue bars), which still retained ≈40 of the wound area. The superior performance of the combined formulation suggests a synergistic effect between PAH_0.24_G_37_ and pEVs, likely due to enhanced delivery and retention of the therapeutic pEVs at the wound site, promoting more efficient tissue repair and regeneration. Altogether, this demonstrated that PAH_0.24_G_37_@pEVs treatment led to superior wound healing, with complete closure observed by day 14, whereas the PBS‐treated wounds showed significant residual wound area, highlighting the inadequacy of the negative control in promoting wound healing.

Compared to the pEVs‐loaded GelAlg@rGO, where ≈5% of the wound area remained open after 14 days, PAH_0.24_G_37_@pEVs treatment proved also to be healable. The PAH_0.24_G_37_@pEVs formulation achieved however complete wound closure within the same timeframe as the pEVs‐loaded GelAlg@rGO hydrogels activated by near‐infrared radiation (NIR) for 5 min at 2 W cm^−2^.^[^ ^14f^ ^]^ The elimination of the need for additional NIR light treatment significantly simplifies wound management and marks a significant step forward in optimizing wound care therapies, ensuring effective healing outcomes with simpler treatment protocols.

**Figure 8:**
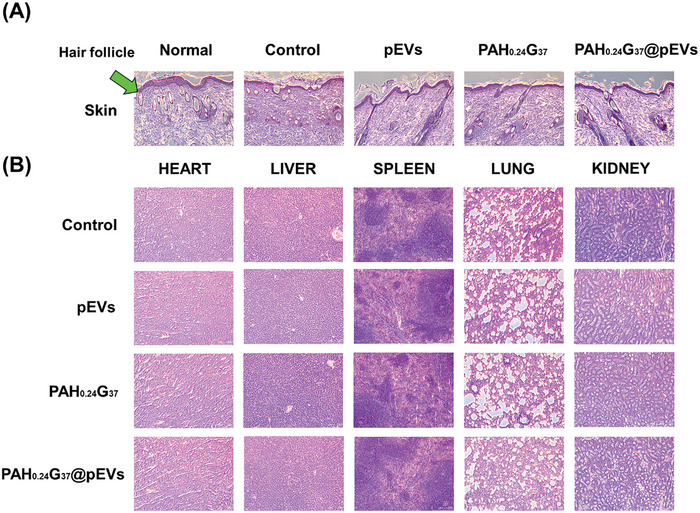
Histological analysis. (A) Haematoxylin and Eosin stained sections of skin tissues and (B) major organs from diabetic rats treated with different formulations. PBS (negative control), pEVs (1 × 10^11^ particles mL^−1^), PAH_0.24_G_37_ and PAH_0.24_G_37_@pEVs (1 × 10^11^ particles mL^−1^). The images show no visible signs of tissue damage or inflammation.

**Figure** **8**A illustrates the histological analysis of skin tissues from diabetic rats after a 14‐day treatment period using Haematoxylin and Eosin (H&E) staining. The PBS‐negative control group displayed minimal follicular regeneration and hair growth, related to poor wound healing and tissue regeneration. This lack of regeneration highlights the challenges in treating chronic diabetic wounds, where impaired healing mechanisms are prevalent. The tissues treated with pEVs (1 × 10^11^ particles mL^−1^) demonstrated moderate regeneration of hair follicles and some degree of epidermal restoration. This observation suggests that pEVs alone have a positive impact on tissue regeneration, although the extent of regeneration was not as pronounced compared to the more advanced treatments. In the group treated with PAH_0.24_G_37_, slight follicular and tissue regeneration were clearly observed, showing that while with PAH_0.24_G_37_ contributes to wound healing, it may require additional factors to achieve optimal results. The most significant changes were noticed in the PAH_0.24_G_37_@pEVs‐treated group, where the skin tissues showed well‐organized granulation tissue, substantial regeneration of hair follicles, and restored squamous epithelium. This suggests that the combined treatment with PAH_0.24_G_37_ and pEVs effectively enhances wound healing by promoting both epidermal restoration and hair follicle regeneration. The structural integrity of the regenerated tissue in this group appears to be comparable to healthy skin, underscoring the potential of PAH_0.24_G_37_@pEVs as a robust treatment strategy for diabetic wounds.

To evaluate the potential in vivo toxicity associated with various treatments, major organs, including the heart, liver, spleen, lungs, and kidneys, were harvested from diabetic rats after a 14‐day treatment period. These organs were subsequently examined histologically using hematoxylin and Eosin (H&E) staining, as depicted in Figure 8B. The results were remarkable; tissue sections from animals treated with PAH_0.24_G_37_@pEVs exhibited no detectable damage or signs of inflammatory lesions in any of the examined organs, highlighting the formulation's safety for therapeutic applications in managing diabetic wounds. Further systemic biocompatibility assessments of the PAH_0.24_G_37_@pEVs were conducted, focusing on the histopathological examination of vital organs. These assessments involved detailed histological analyses of the heart, liver, spleen, lungs, and kidneys across several groups: a normal control group, a diabetic wound group treated with pEVs, and a diabetic wound group treated with PAH_0.24_G_37_ and PAH_0.24_G_37_@pEVs. In all cases, there were no noticeable signs of tissue damage or inflammation, indicating that the PAH_0.24_G_37_@pEVs did not induce any toxic effects on the metabolic or excretory functions of these organs. Together with the H&E staining results, revealing no detectable damage, inflammation, or lesions in any of the examined organs across all treatment groups, including the PAH_0.24_G_37_@pEVs‐treated group, and the absence of histopathological abnormalities in vital organs indicate that the treatments, particularly PAH_0.24_G_37_@pEVs, did not induce systemic toxicity, an essential criterion for any therapeutic application. These findings are especially relevant for diabetic patients, who often experience multiple comorbidities that could be exacerbated by systemic toxicity. Achieving effective wound healing without compromising the health of critical organ systems is a significant advantage. Moreover, the study's results suggest that PAH_0.24_G_37_@pEVs could be safely utilized in a clinical setting for the treatment of chronic diabetic wounds, potentially offering a therapeutic solution that combines efficacy in wound healing with a high degree of safety. This is crucial for developing new treatments aimed at improving the quality of life for diabetic patients by providing a more efficient and safer wound care option.

The comprehensive evaluation confirms that PAH_0.24_G_37_@pEVs possessed excellent biocompatibility, making it a promising translational option for diabetic wound treatment using pEVs from autologous or allogeneic clinical‐grade sources,^[^ ^15^ ^]^ as wound healing enhancers. This is significant as it not only ensures the safety of the hydrogel formulation but also underscores its potential as an effective therapeutic agent in clinical settings. The absence of adverse histological changes in critical organ systems further reinforces the therapeutic viability of PAH_0.24_G_37_@pEVs, suggesting that they can be safely applied without concern for systemic toxicity. These findings are crucial for advancing the clinical development of new treatments for chronic diabetic wounds, offering management that combines efficacy with a high degree of safety.

**Figure 9:**
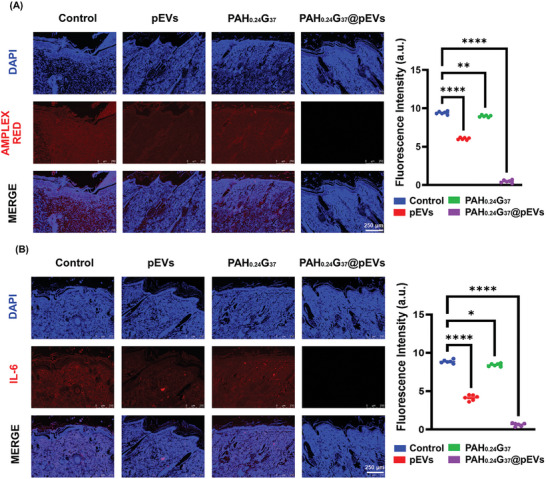
Microscopic evaluation of inflammatory response. Images of (A) Amplex red assay and (B) IL‐6 immunofluorescence staining assay showing reactive oxygen species (ROS) levels in diabetic wounds treated with PBS, pEVs, PAH_0.24_G_37_, and PAH_0.24_G_37_@pEVs. All in vivo experimental procedures were independently replicated six times (*n* = 6). Results are presented as the mean ± standard deviation (SD). One‐way ANOVA (Tukey's multiple comparison test) was employed to assess statistical significance, with significance levels denoted as follows: *p ≤ 0.05, **p ≤ 0.01, ***p ≤ 0.001, and ****p ≤ 0.0001. The number of ROS species (Amplex red) and the inflammatory response (IL‐6) is minimized by the application of the pEVs‐doped hydrogel.

Chronic diabetic wounds frequently persist in a highly inflammatory state, which can complicate and prolong the healing process.^[^ ^24^ ^]^ To evaluate the inflammatory status of such wounds, the Amplex Red assay was utilized to measure reactive oxygen species (ROS) levels (**Figure** **9**A). The PBS control as well as the PAH_0.24_G_37_ groups exhibited relatively high fluorescence signals, indicative of elevated ROS concentrations and ongoing inflammation within the wounds. Indeed, mitochondrial dysfunction leads to the production of ROS and subsequent oxidative damage. Interestingly, the group treated with pEVs showed a moderate reduction in ROS levels, which might indicate that pEVs have an anti‐oxidant nature, consistent with the presence of various platelet antioxidant enzymes such as superoxide dismutase, catalase, piroxyredoxine, and glutathione peroxidase (GPX), including GPX‐4.^[^ ^25^ ^]^ Previous studies have confirmed^[^ ^26^ ^]^ that pEVs can restore impaired mitochondrial function, reduce oxidative stress, and restore cellular metabolism by modulating the sirtuin 1 (SIRT1)‐peroxisome proliferator‐activated receptor gamma coactivator 1α (PGC1α)‐mitochondrial transcription factor A (TFAM) pathway. In rat models, pEVs have been shown to slow the progression of IVDD. Our findings further support that the injection of pEVs holds significant promise as a therapeutic strategy for treating patients with intervertebral disc degeneration (IVDD). PAH_0.24_G_37_@pEVs finally preserved a low‐inflammatory environment. This is particularly beneficial as it facilitates the rapid progression of diabetic wounds from the inflammatory phase to the proliferation and remodeling phases, which are crucial for successful healing. Similarly, IL‐6 immunofluorescence staining (Figure 9B) revealed that while PBS and the PAH_0.24_G_37_groups showed high fluorescence signals, reflecting ongoing inflammation, PAH_0.24_G_37_@pEVs bandages maintained a reduced‐inflammatory environment from the onset of treatment. This is advantageous for the wound healing process as it promotes a quicker transition from the inflammatory phase to the proliferation and remodeling phases.

**Figure 10:**
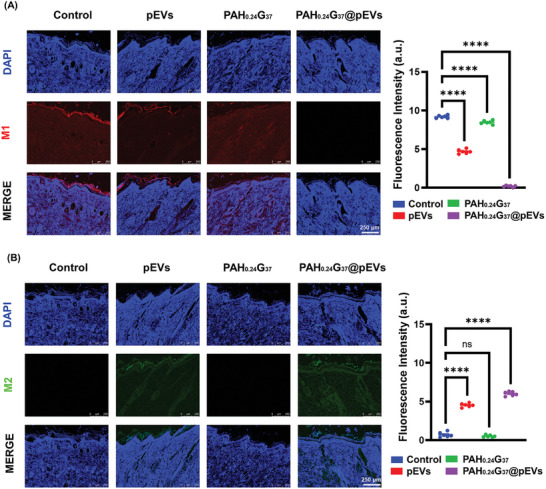
PAH_0.24_G_37_@pEVs impact on macrophage polarization. Immunofluorescence staining of PBS, pEVs, PAH_0.24_G_37_, and PAH_0.24_G_37_@pEVs treated wounds for (A) CD86 to assess M1 macrophage polarization and (B) CD206 for M2 macrophage assessment. All in vivo experimental procedures were independently replicated six times (*n* = 6), and the results are presented as the mean ± standard deviation (SD). The one‐way ANOVA (Tukey's multiple comparison test) was employed to assess statistical significance, with significance levels denoted as follows: *p ≤ 0.05, **p ≤ 0.01, ***p ≤ 0.001, and ****p ≤ 0.0001. The images reveal a greater presence of anti‐inflammatory macrophages (M2) than inflammatory macrophages (M1) when using the pEVs‐doped hydrogel.

Finally, to investigate the potential of PAH_0.24_G_37_@pEVs in influencing macrophage polarization within diabetic wounds, immunofluorescence histological analysis was performed to detect M1 and M2 macrophages using CD86 and CD206 markers, respectively (**Figure** **10**). The results demonstrated a significant M1 to M2 polarization shift in the PAH_0.24_G_37_@pEVs group, indicating an effective macrophage response when compared to the PBS control, and the groups treated with pEVs alone or PAH_0.24_G_37_. This is consistent with in vitro results (see Figure S5, Supporting Information) where similar trends in macrophage polarization were observed, validating the effectiveness of PAH_0.24_G_37_@pEVs in modulating macrophage polarization. A previous study demonstrated that platelet‐derived exosomes inhibited the polarization of M1 macrophages by modulating the NF‐κB and MAPK pathways.^[^ ^27^ ^]^ Additionally, these exosomes influenced the polarization of M2 macrophages by regulating STAT6 phosphorylation. Furthermore, platelet‐derived exosomes promoted the autophagic degradation of NLRP3 by enhancing its ubiquitination, thereby reducing the production of IL‐1β and Caspase‐1.

To further explore the regulatory mechanisms of pEVs on macrophage signaling pathways, Song et al. previously utilized Gene Set Enrichment Analysis (GSEA) to assess alterations in these pathways.^[^ ^28^ ^]^ When comparing a LPS group to a LPS plus pEVs group, enrichment scores for the TGF‐β signaling pathway, ECM receptor interaction, IL‐10 signaling, extracellular matrix organization, and VEGF signaling, indicating an upregulation of these genes. Conversely, the collagen degradation pathway reflected downregulated gene expression. Under inflammatory conditions, these specific gene groups (TGF‐β, IL‐10, VEGF) are known to facilitate the transition from M1 to M2 macrophages, suppress excessive inflammatory responses, enhance angiogenesis, stimulate collagen formation, and reduce collagen breakdown. As a result, pEVs are believed to exert pro‐reparative effects, making them promising candidates for treating diseases characterized by an impaired M1–M2 transition, ultimately aiding tissue repair and regeneration.

**Figure 11:**
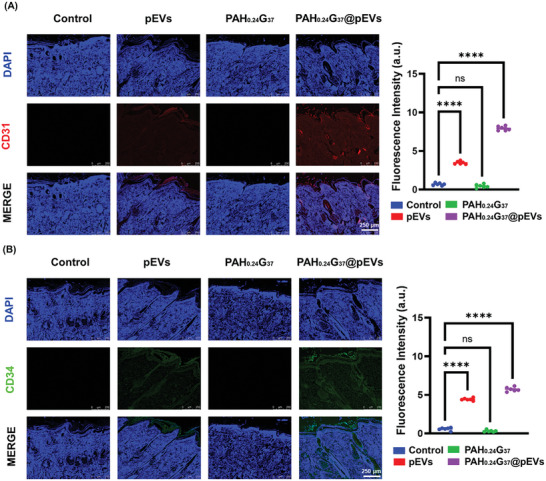
PAH_0.24_G_37_@pEVs enhanced vascular formation and follicle activation. Immunofluorescence staining (A) CD31 and (B) CD34 on wounds treated with PBS, pEVs, PAH_0.24_G_37_, and PAH_0.24_G_37_@pEVs. All in vivo experimental procedures were independently replicated six times (*n* = 6), and the results are presented as the mean ± standard deviation (SD). One‐way ANOVA (Tukey's multiple comparison test) was employed to assess statistical significance, with significance levels denoted as follows: *p ≤ 0.05, **p ≤ 0.01, ***p ≤ 0.001, and ****p ≤ 0.0001. The images reveal the more marked presence of endothelial cells (CD31) and hematopoietic stem cells and vascular progenitor cells (CD34) when using the pEVs‐doped hydrogel.

Finally, the formation of vascular structures could be validated using CD31 and CD34 immunofluorescence (**Figure** **11**), with PAH_0.24_G_37_@pEVs exhibiting notable improvements in enhanced follicle activation and angiogenesis, compared to the other groups. Platelet‐derived exosomes have been reported to effectively promote the proliferation and migration of endothelial cells and fibroblasts, enhancing angiogenesis and re‐epithelialization in chronic wounds.^[^ ^29^ ^]^ Moreover, it was proposed that interfollicular injections of autologous CD34+ cell‐containing PRP preparations exhibit a beneficial therapeutic impact on androgenetic alopecia in both males and females, with no notable substantial adverse effects.^[^ ^30^ ^]^

## 3 Conclusion

The success of pEVs in promoting wound healing depends not only on their bioactivity, but also on their effective delivery and retention at the wound site. It could be shown that the excellent swelling properties of PAH_0.24_G_37_ hydrogel provide a suitable matrix for pEVs loading and delivery, ensuring that the vesicles remain localized at the wound site for a sufficient duration is critical. Using an in vitro wound healing assay based on GFP expressing human adult dermal fibroblast cells, the concentration‐dependent effect of pEVs on wound closure was confirmed. The concentration of pEVs ≥ 3 × 10^9^ particles mL^−1^ showed wound healing rates approaching that of the full medium. In vivo studies demonstrated indeed full wound closure after 14 days when treated at days 0 and 7 with PAH_0.24_G_37_@pEVs bandages loaded with pEVs (1 × 10^11^ particles mL^−1^). The superior therapeutic outcomes observed with PAH_0.24_G_37_@pEVs were ascribed to the improved mechanical properties of the PAH‐G matrix and its excellent swelling capacity. This allows the encapsulation of a large number of therapeutic pEVs and a capacity to avoid pEVs leakage, likely taking place when the pEVs are not loaded in the hydrogel foam. Sustained release over several hours in addition ensured that the pEVs consistently reached the target cells and tissues, optimizing the healing effects. The maintenance of a reduced‐inflammatory environment, as well as M1–M2 macrophage modulation, helped in addressing the complex challenges associated with chronic diabetic wounds and represent a step further into advanced treatment regimens for chronic diabetic wounds. Although the study demonstrated excellent biocompatibility of PAH_0.24_G_37_@pEVs in preclinical models, the long‐term safety of these materials in humans requires thorough investigation. Potential immunogenic responses to pEVs, particularly when derived from allogeneic sources, could pose risks, including unwanted immune reactions or the exacerbation of inflammation in some patients. Extensive safety and toxicology studies are essential to evaluate any potential adverse effects, including the possibility of hypersensitivity or other immune‐related issues in diverse patient populations.

While this study clearly underlines that pEVs‐loaded hydrogels represent an efficient approach to maintaining the biological activity of pEVs, as they are not washed out by the wound exudate and can efficiently manage wound inflammation and accelerate the wound's transition into the proliferation phase, several outstanding questions remain before clinical translation is guaranteed. The therapeutic efficacy of pEVs may be influenced by several factors, including the donor's health status, platelet concentrate preparation methods, and isolation techniques. These variabilities can lead to inconsistencies in pEVs' composition and bioactivity, likely affecting their therapeutic outcomes. Standardization of pEVs production, including rigorous quality control measures, remains crucial to ensure consistent therapeutic effects when applied in a clinical setting. Furthermore, chronic wounds, especially those associated with diabetes, are heterogeneous in nature, with varying degrees of severity, underlying pathologies, and patient‐specific factors, such as age, comorbidities, and nutritional status. These factors can influence the wound‐healing process and may affect the clinical efficacy of the proposed PAH_0.24_G_37_@pEVs. Personalized approaches that consider these variables may be needed to optimize treatment outcomes, complicating the development of a one‐size‐fits‐all therapeutic solution. Another main challenge lies in the duration of efficacy and storage of hydrogels loaded with pEVs. These aspects will be the subject of future studies.

While pEVs, as cell‐free therapeutics, present limited ethical or safety concerns compared to traditional cell‐based therapies, when obtained from autologous origin or healthy donors, no pEV‐based hydrogel product is currently FDA‐approved. Standardized pEV production protocols and validation processes are needed to achieve the regulatory approvals required for market approval and further clinical translation. Cost considerations and scalability are additional challenges that will guide the future of pEVs‐hydrogel bandages. Introducing a new therapeutic approach into established clinical practice involves not only the demonstration of efficacy and safety but also ensuring that healthcare providers are adequately trained and equipped to administer the treatment. The integration of PAH_0.24_G_37_@pEVs into wound care protocols will require careful consideration of existing clinical workflows, training for clinicians, and possibly the development of new guidelines for the management of chronic wounds. In addition, to reach the translational research stage, studies of the biodistribution of pEVs and their effects on other organs will need to be undertaken. While promising short‐term results have been reported here, no long‐term effects of repeated hydrogel applications have been conducted so far. The possibilities of chronic inflammation, fibrosis, or other adverse reactions over time cannot be ruled out even though gelatin‐based hydrogels have long been known to be non‐toxic and fully biocompatible. Glutaraldehyde, like all aldehydes, is a very reactive species and should be inert after some hours when crosslinked. Extensive washing steps ensured in addition that all non‐crosslinked molecules were removed from the hydrogel.

Additionally, the safety of using pEVs loaded into hydrogels for treating wounds is supported by various factors. First, the incorporation of pEVs within hydrogels limits the possibility that they would leak into the blood system and target other organs. Second, our studies have shown that pEVs are nontoxic and quickly internalized by several cell types present in their immediate environment,^[^ ^31^ ^]^ limiting the fact that they may target other organs under topical administration as is the case for wound healing. Third, the p‐EVs used in this study are generated from platelet concentrates which are an essential medicine already widely applied in the clinic for intravenous administration. Such platelet concentrates contain a high number of p‐EVs which are either collected from the blood circulation or released from platelets in vitro during the storage period.^[^ ^15^ ^]^ This implies that p‐EVs are already administered in an unpurified form to patients when they receive PC infusions.

In conclusion, while PAH_0.24_G_37_@pEVs show great potential for enhancing wound healing, several challenges must be addressed to translate these preclinical findings into clinically viable therapy. Future research should focus on addressing these limitations through comprehensive safety and efficacy studies, optimizing pEVs production and delivery, and developing strategies to ensure the economic feasibility and accessibility of this promising treatment.

## 4 Experimental Section

### Chemicals

Gelatine from bovine skin (G9382, 225 Bloom, Type B), glutaraldehyde (G6257), poly(allylamine hydrochloride) (PAH, 283215, Mw≈17500), sodium chloride (C57653), sodium hydrogenocarbonate (S5761), potassium chloride (P3911), calcium chloride (C1016), sodium hydroxide (30620), bovine serum albumin (BSA, 05482), resazurin sodium salt (R7017), trypsin‐EDTA solution (T3924), trypsin inhibitor from Glycine max (soybean) (T6414) were purchased from Merck. Hank's balanced salt solution (Thermo Scientific™ 88284), phosphate buffer saline (PBS) pH 7.4 (Gibco™ 10010056) were procured from Fisher Scientific. All reagents were used without purification. Platelet concentrates were obtained from healthy volunteer donors at the French National Blood Service in Saint‐Etienne, France, or Taipei Blood Center, Taiwan. FibroLife fibroblast serum‐free medium complete kit (LL‐0001) and FibroLife fibroblast basal medium (LM‐0001) were purchased from Cellsystems GmbH (Troisdorf, Germany). GFP‐expressing human adult dermal fibroblast cells (HDFCs‐adGFP) were obtained from PELOBiotech GmbH (Planegg/Martinsried, Germany). The type I ultrapure water used throughout the experiments was purified with a purification system Arium® comfort I from Sartorius (resistivity = 18.2 MΩ.cm).

### Synthesis of Gelatine Hydrogel Foams G_17_, G_27_, G_37_

Gelatine (1.15, 1.82, or 2.5 g) in sodium acetate (50 mM, pH 5–5.5) was heated until its complete dissolution. Then, 1 mL of glutaraldehyde aqueous solution (26.47 mM) was added dropwise and immediately 0.5 mL of NaHCO_3_ (89 mM) was injected into the mixture. The formed gelatine hydrogel foam was poured into a Petri dish and frozen at −20 °C for 10 min. The hydrogels were then placed in pure water for 17 h to remove impurities and excess starting materials. The washed hydrogel foams were finally dried at room temperature for 3 days and stored in Falcon^TM^ tubes for future experiments.

### Synthesis of PAH_x_G_37_ (x = 0.12, 0.24, 0.36) Hydrogel Foams

5.18 mL of poly(allylamine)hydrochloride (PAH, 0.155, 0.310, or 0.429% w/v) in sodium acetate (50 mm, pH 5–5.5) was heated at 80 °C for 5 min. Gelatine (2.5 g) was added, and the resulting solution was heated until complete gelatine dissolution. Then, 1 mL of glutaraldehyde aqueous solution (26.47 mM) was added dropwise and immediately 0.5 mL of NaHCO_3_ (89 mM) was injected into the mixture. The formed hydrogel foam was poured into a Petri dish and frozen at −20 °C for 10 min. The hydrogels were then placed in pure water for 17 h to remove impurities and excess starting materials. The washed PAH‐G hydrogel foams were finally dried at room temperature for 3 days and stored in Falcon^TM^ tubes for future experiments.

### Swelling and Degradation Experiments

Dry hydrogel foams (and non‐foams) were immersed in water and the increase in mass was monitored. For this, hydrogels were cut into disks (≈0.5 cm in diameter and 1 cm thick) with an average weight of 145 ± 38 mg. Data were obtained in triplicate. For degradation experiments, mass loss of water‐loaded hydrogel foams (and non‐foams) was monitored by weighing the hydrogels until the complete degradation at room temperature.

### Human Platelet‐Derived Platelet Extracellular Vesicles (pEVs)

pEVs were extracted from lysates made from the clinical‐grade platelet concentrate units. The protocol used was approved by the French ethics boards (CODECOH DC‐2022‐5313). These platelet units were frozen and stored at −80 °C until further processing.

### Serum‐Converted Platelet Lysate Fabrication

50 mL of pooled platelet concentrates were mixed with sterilized zirconium oxide or glass beads (25 g) and 0.22 µm‐filtered (EMD Millipore) CaCl_2_ (23 mM) and left for 60 min until fibrinogen conversion into fibrin and precipitation was observed. The supernatant was centrifuged and concentrated with Amicon^®^ Ultra‐15 (RC, MWCO 100000 Da) at 6000 times gravity for 30 min at 22 ± 2 °C on a Heraeus Biofuge Stratos Centrifuge from Thermo Scientific^TM^. The pEVs concentrate lysate was aliquoted in 0.5 mL fractions and stored at −80 °C.

### Purification by Size Exclusion Chromatography (SEC)

The concentrated pEVs were purified using a Sepharose CL‐2B column (Sigma‐Aldrich, 10 mL) as described before.^[^ ^22^ ^]^ The column was equilibrated with 50 mL of PBS containing 0.32% of filtered tri‐sodium citrate solution (0.22 µm filter, EMD Millipore). One aliquoted fraction (0.5 mL) was injected into the column. The eluate was collected into fractions of 0.5 mL immediately after fraction deposition. The first 10 fractions were harvested and analyzed by NTA to detect the presence of pEVs. The column was then washed with 15 mL of PBS (0.32% tri‐sodium citrate), 50 mL of NaOH (0.5 M), 15 mL of PBS (0.32% tri‐sodium citrate), 50 mL of Milli‐Q® water, and 50 mL of degassed ethanol solution (20% v/v) for further cycles (maximum 3–5 times).

### Load and Release Experiments—Fluorescent Labelling of pEVs

pEVs were marked with 3% v/v of 3,3‐dioctadecyloxacarbocyanine perchlorate (DiO) solution (1.4 mg mL^−1^ H_2_O/MeOH (99/1)) during 1 h at 4 °C in the dark. G‐25 desalting spin columns (www.advansta.com) were centrifuged 6 times at 800 × g for 1 min with the MicroCL 21R Centrifuge (ThermoFisher, Osterode am Harz, Germany), one time to remove the storage solution and 5 times with PBS. Then, the DiO‐loaded pEVs solution was centrifuged one time at 1500 × g for 2 min (180 µL) to separate the fluorescent labeled pEVs from the free dye.

### Load and Release Experiments—pEVs Loading

PAH_0.24_G_37_ hydrogel foams (140 ± 16 mg) were placed into 400 µL of DiO‐labeled pEVs previously extracted by Zeba spin desalting columns during 3.5 h at 4 °C. The hydrogels were washed by immersion for 10 min at 4 °C into ultrapure water before use.

### Load and Release Experiments—pEVs Release

PAH_0.24_G_37_ hydrogel foams containing pEVs ((6.4 ± 0.27) × 10^10^ particles mL^−1^) were immersed in 1 mL of PBS and incubated at 37 °C for 17 h and the release of pEVs was followed by F‐NTA in fluorescence mode (488 nm laser and LP cut‐off 500 nm).

### Bicinchoninic Acid Assay (BCA) Test for Protein Quantification

The protein quantification was determined by bicinchoninic acid assay test with BCA Protein Assay Kit (EMD Millipore); 15 µL of each pEVs solution were diluted in 135 µL of 4% CuSO_4_ aqueous solution. The calibration curve was prepared by adding 0, 5, 7, 10, and 15 µL of BSA standard solution in 4% CuSO_4_ solution to obtain 150 µL in a 96‐well plate. The plate was incubated at 37 °C during 30 min and read at 562 nm with Cytation™ 5 Cell Imaging Multi‐Mode Reader (BioTek Instruments SAS, France).

### Characterization—Rheological

Hydrogels were cut into disks of ≈2.9 cm in diameter and 2–3 mm thick and stored 1 day in humid conditions before being analyzed with a rheometer MCR302e (Anton Paar, Les Ulis, France). The temperature sweep experiments were performed with a gap of 1.5 mm and a 2.5 cm diameter plan (PP25) from 25 to 60 °C at 1 Hz.

### Characterization—Scanning Electron Microscopy (SEM)

The hydrogels were freeze‐dried just after the synthesis. The samples were frozen during 3 h at −80 °C and freeze‐dried all night (0.01 mbar, −60 °C) with the Alpha 2–4 LSCbasic (CHRIST Company, Osterode am Harz, Germany). The sponge samples were sectioned with a razor blade as reported previously and coated with gold. Scanning electron microscopy (SEM) was performed with a FEI Quanta FEG 250 microscope (FEI Company, Eindhoven, The Netherlands) with an accelerating voltage of 5 kV.

### Characterization—Nanoparticle Tracking Analysis (NTA)

pEVs concentrations and sizes were determined using the ZetaView® MONO (PMX‐130, Particle Metrix, Germany) using an optimized protocol for EVs developed by Particle Metrix.

### Characterization—Determination of Morphology of EVs‐SCPL

The morphology of serum‐converted platelet lysate (SCPL) and SEC‐purified SCPL‐EVs samples was checked using cryo Electron Microscopy (cryoEM). 4 µL of SCPL (obtained as described above) and SCPL‐EVs (formulated in the PBS buffer used to equilibrate the SEC column) were applied onto distinct 200‐mesh holey carbon film. The film was previously glow‐discharged for 20 s to clean the surface and enhance its hydrophilicity. The film was then blotted for 3 s in 100% humidity at 4 °C and stored in liquid nitrogen until imaging. The EV samples were loaded onto three grids and vitrified using the FEI Vitrobot 2.

### Biological Assays—Metabolic Activity Tests

Before the assay, HDFCs‐adGFP cells were seeded at a density of 1 × 10^4^ cells/well in a 96‐well plate and grown for 24 h. The dried hydrogels (163 ± 17 mg) were first immersed in basal fibroblast medium (1 mL) for 3.5 h. They were removed and then extracted in another vial tube with 1 mL of complete fibroblast medium for 2 h. Then 200 µL of the pure extracts and control medium were incubated in a 96‐well plate containing cells and left 24 h. The cell layers were washed and 100 µL of resazurin salt solution (11 µg mL^−1^ in complete fibroblast medium) was added to each well and the plate was incubated for 4 h. The fluorescence intensity was measured at 593 nm (20 nm bandwidth) with an excitation at 554 nm (18 nm bandwidth) by the Cytation™ 5 Cell Imaging Multi‐Mode Reader (BioTek Instruments SAS, France). As untreated control, fresh complete fibroblast medium alone was used. Each condition was replicated three times and the mean fluorescence value of control cells was taken as 100% metabolic activity.

### Biological Assays—Dead Cell Visualization

In the same way as metabolic activity tests, the cells were seeded at a density of 0.7 × 10^4^ cells/well and grown for 24 h. Cells were directly stained with propidium iodide (PI) solution after 24 h exposure to the extracts. Propidium iodide (3 µg mL^−1^) was added to each well and the cells were incubated at room temperature for 15 min in the dark. The cells were then visualized using a Cytation™ 5 Cell Imaging Multi‐Mode Reader (BioTek Instruments SAS, France) at 2.5× magnification (2.75× eff.) with a Zeiss Plan Fluorite WD 6.3 mm / NA 0.12 objective (Agilent 1220539) and the corresponding excitation and emission filters (GFP filter: 469‐35/525‐39 nm for GFP expressing cells and RFP filter: 531–40/593‐40 nm for propidium iodide). 0.25% Triton^TM^ X‐100 was used as a positive control for dead cells.

### Biological Assays—Macrophage Polarization In Vitro

Fluorescence microscopy (Leica, Germany) was used to evaluate the polarisation of macrophages in RAW264.7 cells. The cells were obtained from the American Type Culture Collection (ATCC, Manassas, VA, USA). The cells were exposed to 100 ng/mL lipopolysaccharide (LPS) for 24 h and then treated with previously dialyzed culture media (PEV, PAH0.24G37, PAH0.24G37@pEVs) based on experimental groups for an additional 72 h. Following treatment, RAW264.7 cells were rinsed with a buffer solution at a pH of 7.4 and then immobilized in a 4% paraformaldehyde solution for a duration of 30 min. The cells were subsequently treated at a temperature of 4 °C for a duration of 1 h with either FITC‐conjugated anti‐CD206 (M2) antibody or APC‐conjugated anti‐CD86 (M1) antibody (BioLegend, San Diego, CA, USA) in order to facilitate subsequent analysis.

### Wound Healing Assay using Fluorescence Kinetic Imaging

A full step‐by‐step protocol for wound healing assay had been deposited in the protocols repository.^[^ ^32^ ^]^ Briefly, HDFCs‐adGFP cells were seeded at a density of 5 × 10^4^ cells/well in a 96‐well plate and grown for 24 h before assay. Reproducible scratch wounds in cell monolayers grown in microplates were created using sterile 200 µL pipette tips. The cell monolayers were washed with fibroblast basal medium. Fresh basal medium (200 µL) supplemented with mitomycin C (1 µg mL^−1^) that contains the pEVs at various concentrations was added before to start the kinetic. During 30 h, the wound width and the wound confluence were analyzed every hour using fluorescence imaging. The images were recorded using fluorescence microscopy (465 nm LED cube and GFP filter set consists of the EX 469/35 nm, EM 525/39 nm, and dichroic mirror 497 nm filters) with 2.5× objective (Zeiss Plan Fluorite, 2.75x eff., Agilent 1220539) using a Cytation™ 5 Cell Imaging Multi‐Mode Reader (BioTek Instruments SAS, France). During the experiment, cells were maintained at 37 °C and 5% CO_2_ using a CO_2_ controller (Bio‐Tek / Agilent 1210008). Each condition was replicated four times and the maximum wound healing rate was automatically calculated (mean ± SD) using Gen5 Image+ software (version 3.10.06).

### Wound Healing Assay using Streptozotocin (STZ)‐induced Diabetic Wound Model

The Animal Ethics and Use Committee of Taipei Medical University granted approval for all procedures involving animals in this study (LA^[^ ^32^ ^]^C2023‐0083 and LAC 2023‐0316). Wistar rats, weighing between 200 and 240 g (8 weeks old), were sourced from Bio‐LASCO, Taipei, Taiwan, and allowed a 1‐week acclimatization period with access to food and water ad libitum. Following the protocols described in previous studies,^[^ ^14^ ^]^ chronic full‐thickness dorsal skin wounds were induced in diabetic rats. Initially, all rats received intraperitoneal injections of streptozotocin (STZ) at a dose of 40 mg kg^−1^ body weight per day for five consecutive days. Fasting blood glucose levels were subsequently measured using a Roche blood glucose meter (Taiwan). Rats exhibiting fasting blood glucose levels exceeding 250 mg dL^−1^ were selected for the creation of full‐thickness wounds.

One week after confirming hyperglycemia, full‐thickness skin defects with a diameter of ≈8 mm were created on the back of the diabetic rats using a sterile biopsy punch, under anesthesia administered via 3% isoflurane inhalation. Six rats per condition (diabetic wound, diabetic wound + pEVs, diabetic wound + hydrogel, and diabetic wound + hydrogel + pEVs) were used with 2 wounds per rat for a total of 48 wounds. The wounds were treated topically with PBS, pEVs (1 × 10^11^ particles mL^−1^), PAH_0.24_G_37_ (50 mg), and PAH_0.24_G_37_@pEVs (50 mg and 1 × 10^11^ pEVs particles mL^−1^). These wounds underwent treatment with the different bandages on days 0 and 7 of the experiment and the bandages were kept for 7 days. Digital photographs of the wound sites were captured on days 0, 7, and 14 post‐wounding, and the percentage of remaining wound area was quantified using ImageJ software, calculated as ((wound area at various time points) / (wound area on day 0)) × 100%.

On the 14th day post‐wounding, the treated diabetic rats were euthanized, and organs and major tissues including the heart, liver, spleen, lungs, and kidneys were collected and fixed in 4% paraformaldehyde. These tissues were then embedded in paraffin and sectioned to ≈5 µm thickness for routine histological analysis. The sections were stained with hematoxylin and eosin (H&E) for microscopic examination.

For immunofluorescence analysis, skin sections were first blocked with 1% BSA and 0.2% Triton^TM^ X‐100 for 1 h, followed by overnight incubation at 4 °C with fluorescent‐labeled primary antibodies, including anti‐CD86, anti‐CD206, anti‐HSP, anti‐CD31, and anti‐CD34 antibodies. After thorough washing with PBS, the sections were counterstained with 4′,6‐diamidino‐2‐phenylindole (DAPI) for 3 min at room temperature. The immunofluorescence was assessed using a fluorescence microscope (scale bar: 250 µm) and analyzed with ImageJ software.

### Wound Healing Assay using Streptozotocin (STZ)‐induced Diabetic Wound Model—Statistical Evaluation

All in vivo experimental procedures were independently replicated six times (*n* = 6). The collected data were systematically recorded and prepared for analysis, ensuring consistency and accuracy in the measurements. The data were analyzed using one‐way or two‐way analysis of variance (ANOVA), a statistical technique used to assess the interaction between two independent variables and their effect on a dependent variable. GraphPad Prism software (GraphPad Software, La Jolla, CA, USA) was utilized for all statistical analyses. Data were processed to calculate the mean and standard deviation (SD) for each experimental group. The significance levels were set as follows:*p ≤ 0.05,**p ≤ 0.01,***p ≤ 0.001,****p ≤ 0.0001.

## Conflict of Interest

The authors declare no conflict of interest.

## Author Contributions

F.B. engineered hydrogel foam, loading and release of pEVs, assessment of in vitro biocompatibility, and conducted wound scratch assays. A.B. performed wound scratch assays. A.N.E. prepared pEVs and loaded them into bandages for in vivo experiments. J.C.Y. considered hydrogel aspects. J.R. and S.M. engineered hydrogel foam and contributed to writing, reviewing, and editing. T.B. provided supervision, reviewed, edited, and secured funding. R.B. characterized the hydrogel and participated in reviewing and editing. S.S. conceptualized the project, secured funding, provided supervision, and contributed to writing. E.Y.C. conducted in vivo experiments and contributed to writing.

## Supporting information



Supporting Information

## Data Availability

The data that support the findings of this study are available from the corresponding author upon reasonable request.
