# Supplementary material for: Platelet Extracellular Vesicles Loaded Gelatine Hydrogels for Wound Care
Source: Adv Healthc Mater. 2024 Oct 25;14(1):2401914. doi: 10.1002/adhm.202401914 (PMC11694086; doi:10.1002/adhm.202401914)
Supplement: Supplementary file 1 — Supporting Information [file ADHM-14-0-s001.docx]

**SUPPORTING INFORMATION**

**Platelet extracellular vesicles loaded gelatine hydrogels for wound care**

Florence Back,^1*^ Alexandre Barras,^1^ Ariunjargal Nyam-Erdene,^2^ Jen-Chang Yang,^2,3^ Sorin Melinte,^4^ José Rumipamba,^4^ Thierry Burnouf,^2,5^ Rabah Boukherroub,^1^ Sabine Szunerits,^1,6*^ Er-Yuan Chuang,^2,5*^

*^1^Univ. Lille, CNRS, Univ. Polytechnique Hauts-de-France, UMR 8520 - IEMN, F-59000 Lille, France*

*^2^International Ph.D. Program in Biomedical Engineering, College of Biomedical Engineering, Taipei Medical University, Taipei 11031, Taiwan*

*^3^ Graduate Institute of Nanomedicine and Medical Engineering, College of Biomedical Engineering, Taipei Medical University, Taipei Medical University, Shuang‐Ho Campus, New Taipei City, 2351, Taiwan*

*^4^ Université catholique de Louvain, ICTEAM, Louvain-la-Neuve, Belgium*

*^5^Graduate Institute of Graduate Institute of Biomedical Materials and Tissue Engineering, , College of Biomedical Engineering, Taipei Medical University, Taipei Medical University, Shuang‐Ho Campus, New Taipei City, 2351, Taiwan*

*^6^ Laboratory for Life Sciences and Technology (LiST), Faculty of Medicine and Dentistry, Danube Private University, 3500 Krems, Austria*

**Corresponding authors:** [florence.back@univ-lille.fr](mailto:florence.back@univ-lille.fr) **(FB), sabine.**[szunerits@univ-lille.fr](mailto:szunerits@univ-lille.fr) **(SS),** [eychuang@tmu.edu.tw](mailto:eychuang@tmu.edu.tw) (E-YC)


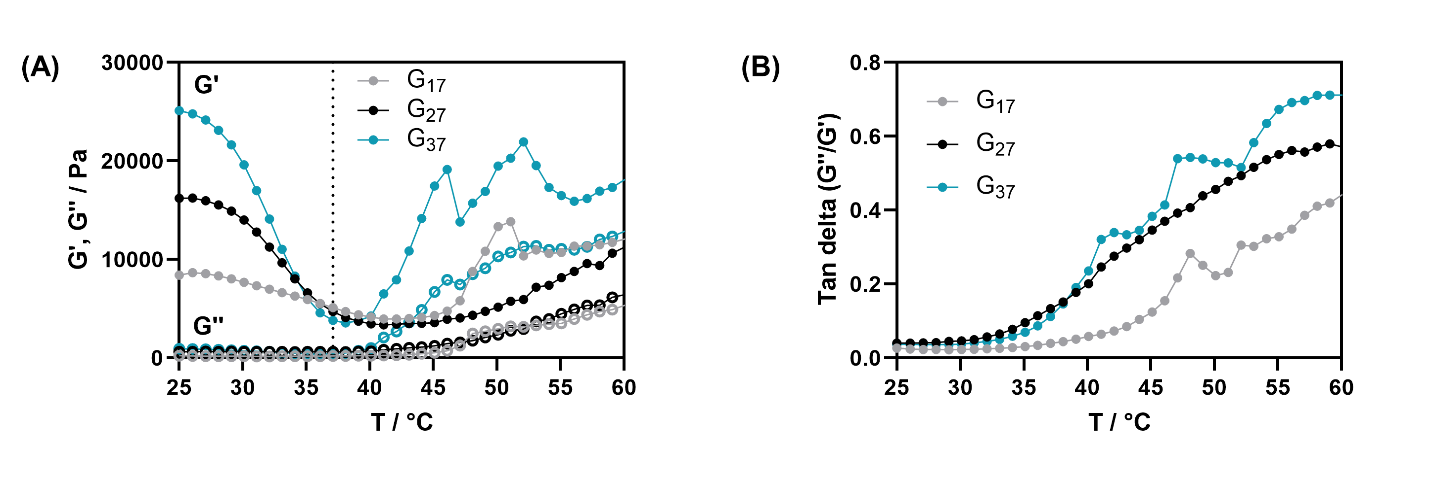


**Figure S1. Mechanical characterization of different gelatine hydrogel foams. (A)** Storage (G’) and loss moduli (G”). **(B)** Tan delta (G”/G’) of gelatine hydrogel foams: gelatine (17%, 27%, 37% w/v) in the presence of glutaraldehyde (26.47 mM) and NaHCO_3_ (89 mM); experimental conditions; 1 Hz, 1% strain, 1.5 mm gap, 1 °C min^-1^.


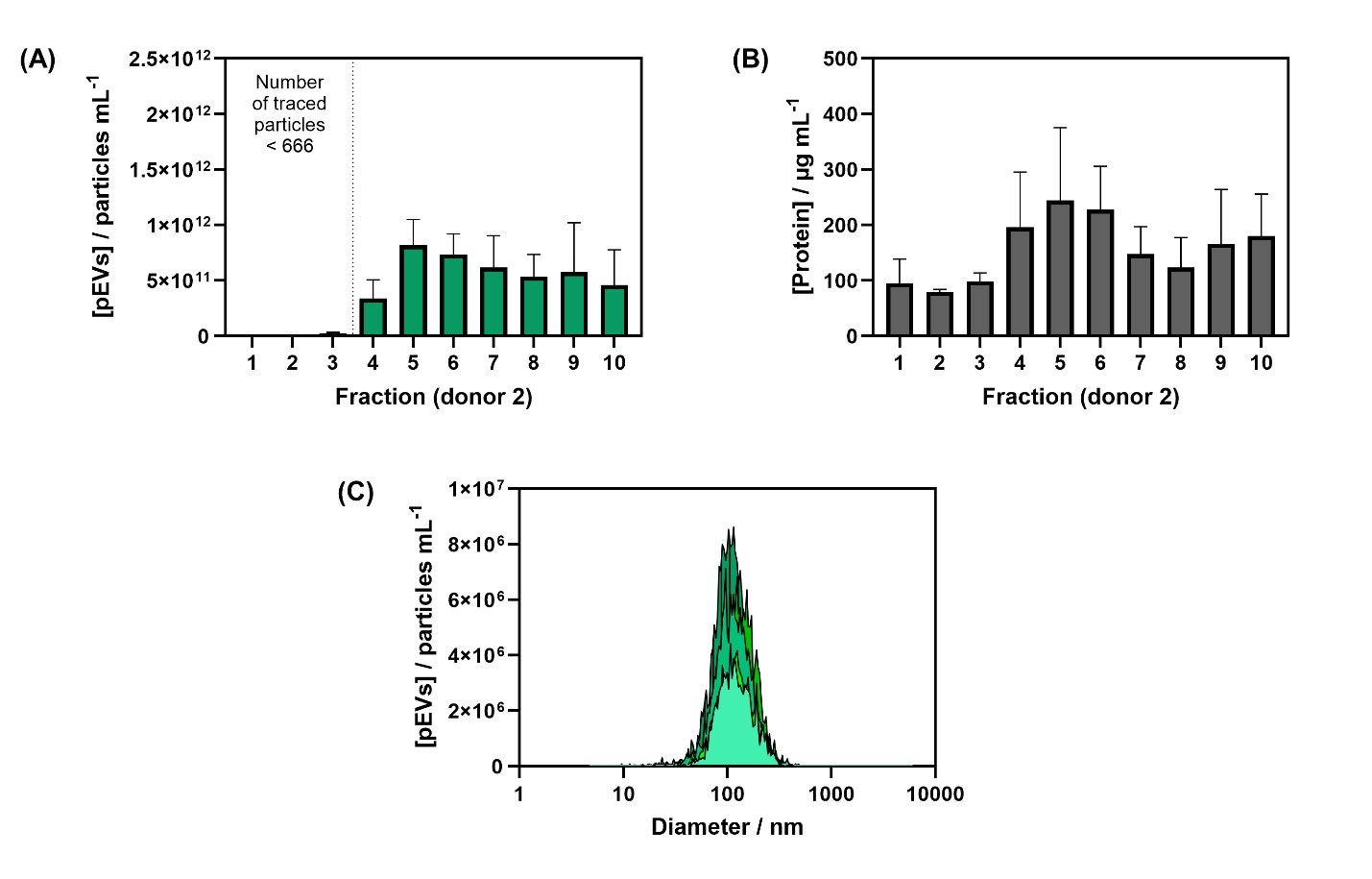


**Figure S2. pEVs characterization of donor 2.** **(A)** Concentration of pEVs as determined by NTA in first 10 fractions collected after column purification. Data are represented as mean ± SD, with n = 5 replicates. **(B)** Total protein concentration in the different fractions using BCA protein assay. Data are represented as mean ± SD, with n = 5 replicates. **(C)** pEVs size distribution of fraction 5 (donor 2, 5 different extractions).


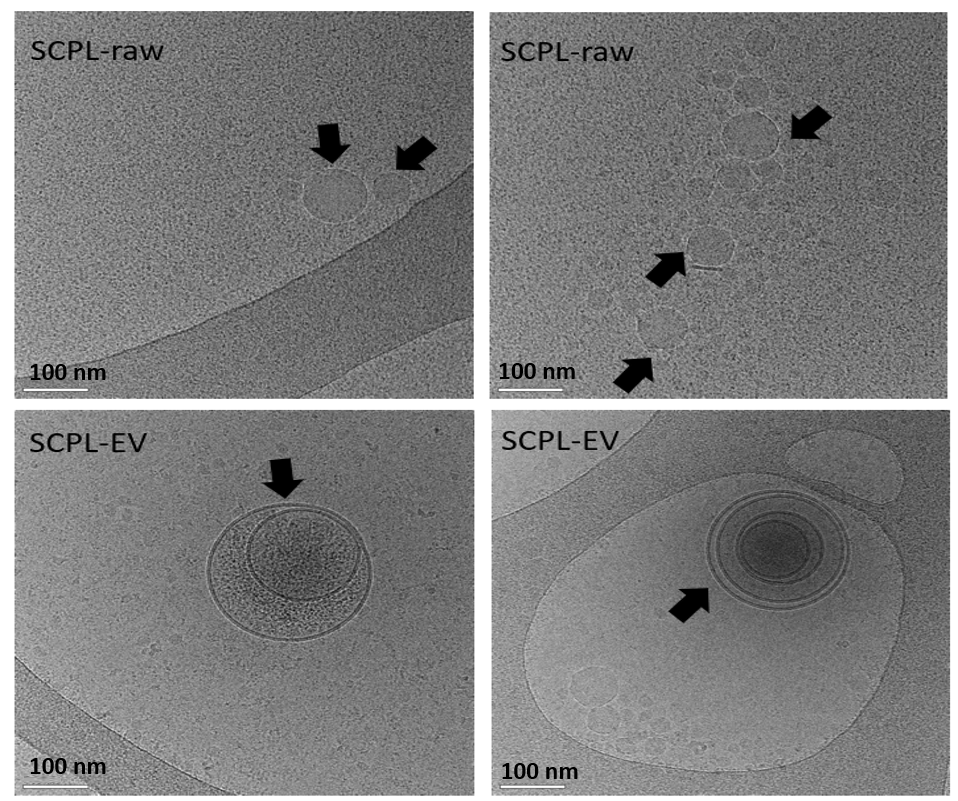


**Figure S3: Cryogenic electron microscopy (cryo-EM) images:** (upper part) serum-converted platelet lysate EVs (SCPL-EVs). (lower part) SEC-purified SCPL-EVs samples


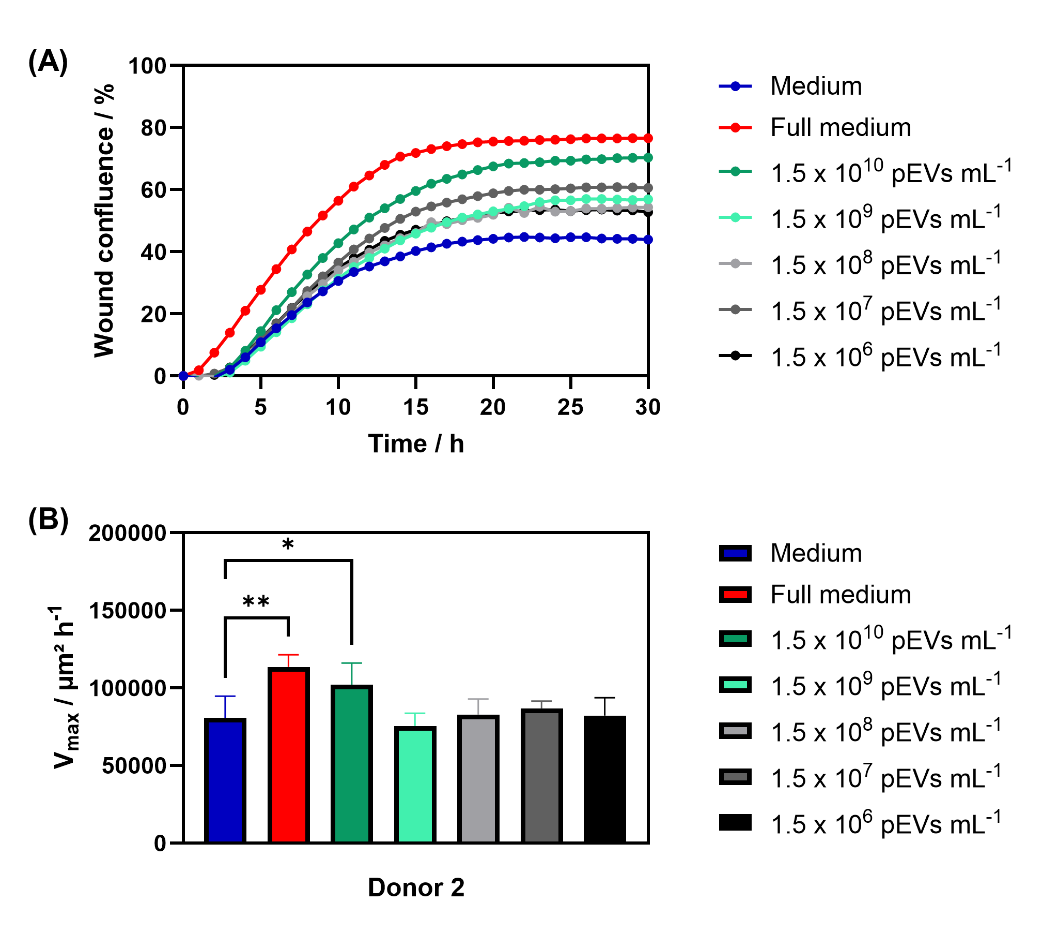


**Figure S4. Effects of donor 2 on wound closure.** **(A)** Wound confluence during 30 h of treatment of HDFCs-adGFP with medium (blue), full medium (red) and 5 different pEV concentrations. Data are represented as mean (without showing SD), with n = 4 replicates. **(B)** Influence of pEV concentration on maximum wound healing rate. Values represent the mean ± SD (n = 4). Ordinary one-way ANOVA with Dunnett’s multiple comparisons test was used to analyze results. Statistically significant differences were considered for **p* ≤ 0.05, ***p* ≤ 0.01, ****p* ≤ 0.001 and *****p* ≤ 0.0001.


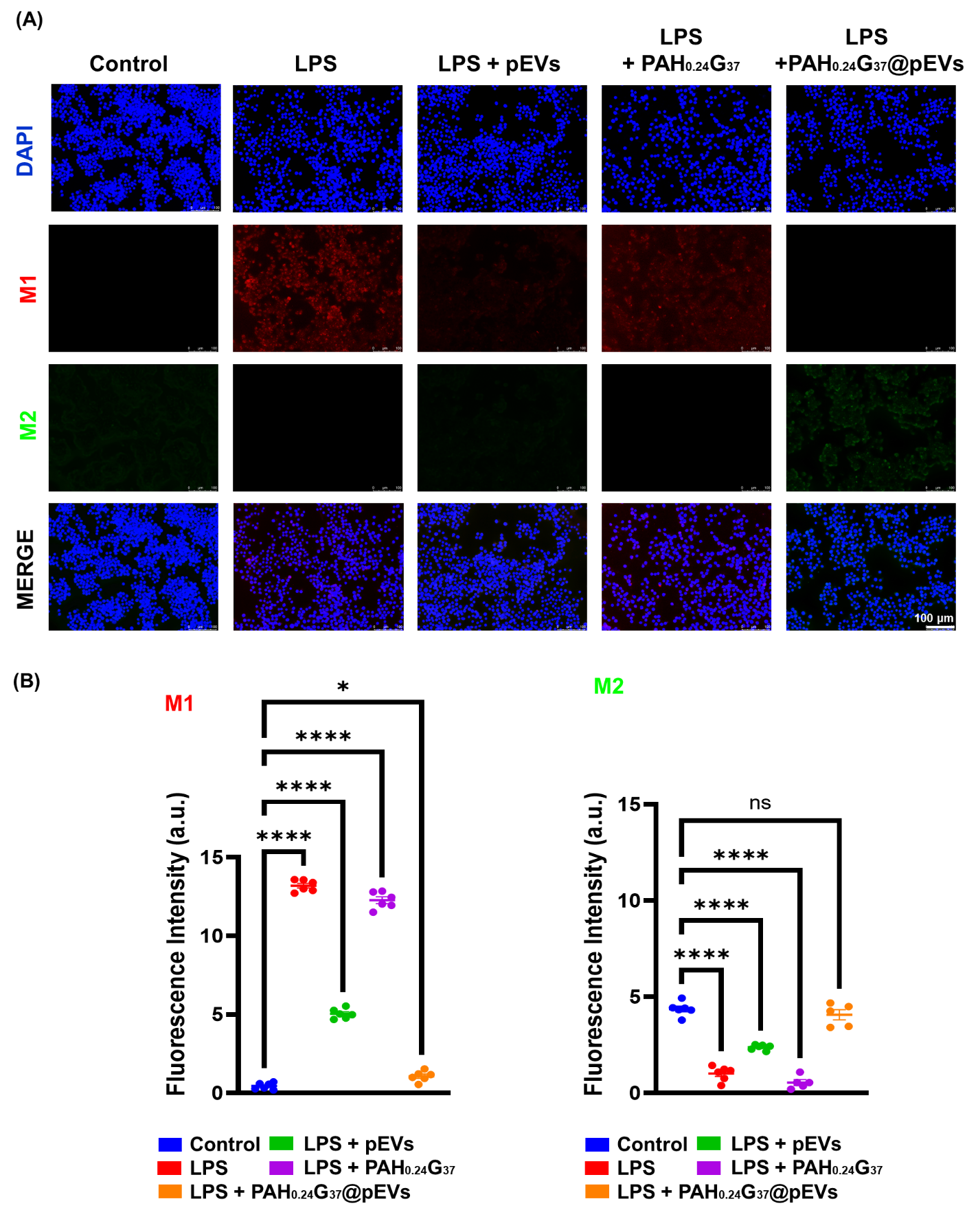


**Figure S5. *In vitro* M1/M2 macrophage polarization investigation: (A)** Immunofluorescence staining images. (**B**) Asessement of M1 macrophage polarization via CD86 and M2 macrophage polarization via CD206. In the control group (PBS) , a minimal presence of M2 macrophages was observed, indicating a baseline state. The LPS group predominantly exhibited M1 macrophages, consistent with a pro-inflammatory response. The LPS + pEVs group demonstrated a mixed polarization with the presence of both M1 and M2 macrophages. The LPS + PAH_0.24_G_37_ group also showed a predominance of M1 macrophages, reinforcing an inflammatory response. In contrast, the LPS + PAH_0.24_G_37_@pEVs group showed a shift towards M2 macrophages, indicating an anti-inflammatory or tissue repair phenotype. Values represent the mean ± SD (n = 6). Ordinary one-way ANOVA with Dunnett’s multiple comparisons test was used to analyze results. Statistically significant differences were considered for **p* ≤ 0.05, ***p* ≤ 0.01, ****p* ≤ 0.001 and *****p* ≤ 0.0001.
